# Supplementary figures and images for: Causal Effect Estimation With TMLE: Handling Missing Data and Near Violations of Positivity
Source: Biom J. 2026 Apr 24;68:e70134. doi: 10.1002/bimj.70134 (PMC13109636; doi:10.1002/bimj.70134)

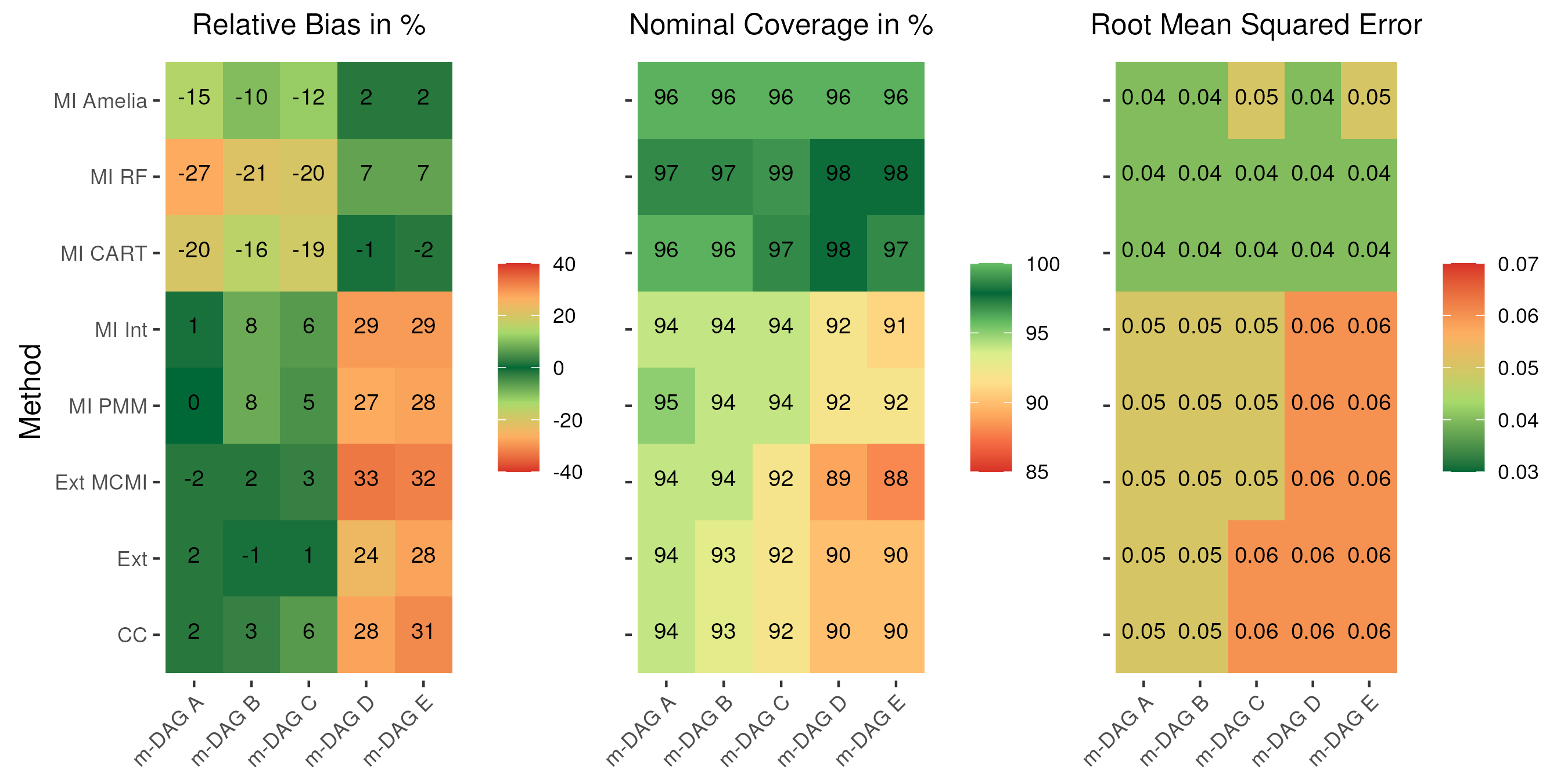

Supplement: Supplementary file 1 — Supporting File: bimj70134‐sup‐0001‐DataCode.zip. [file BIMJ-68-e70134-s001.zip › MissingDataTmle/Simulation/design-based/Results/reference/full/figures/Figure_6.png]

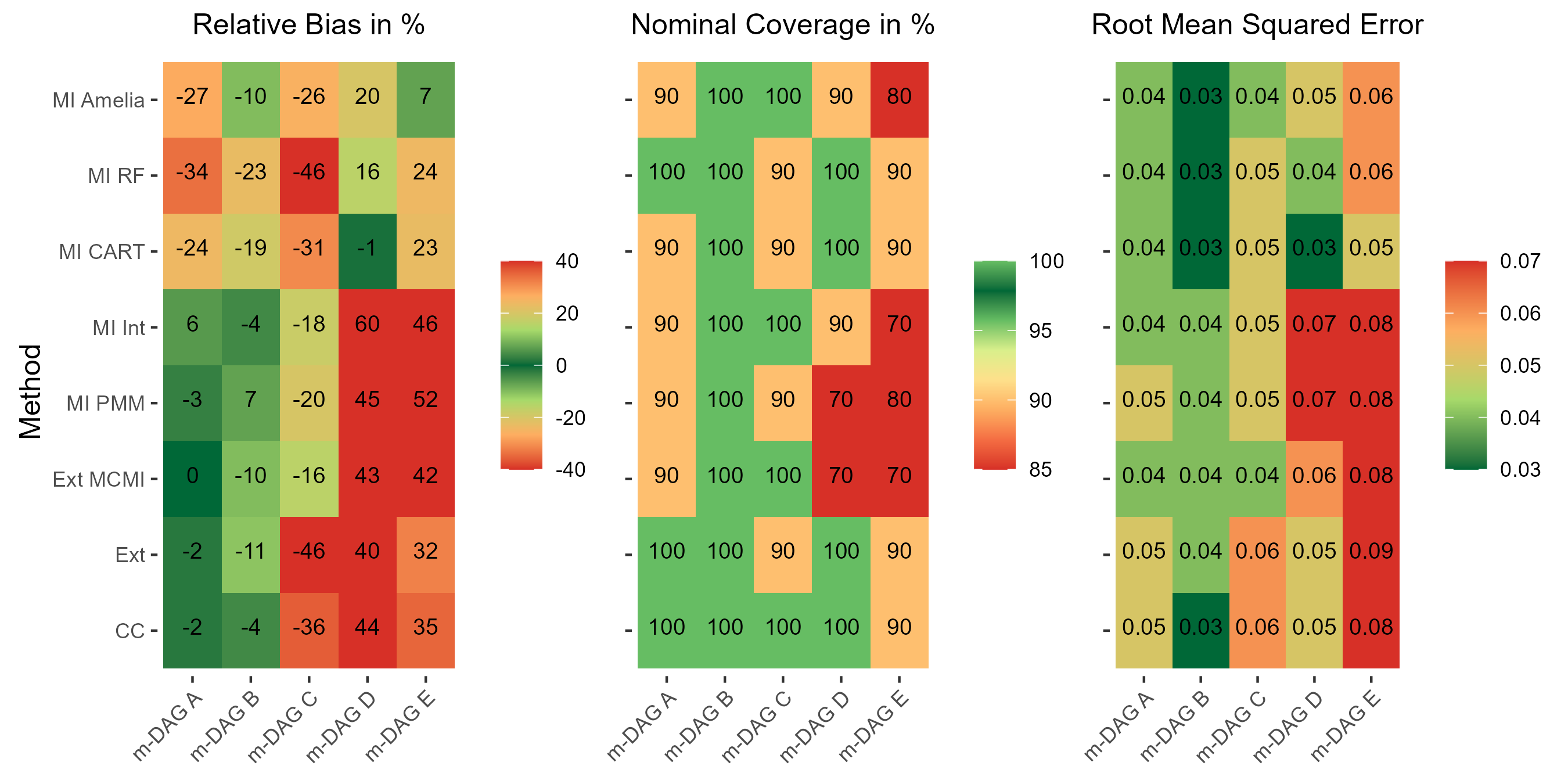

Supplement: Supplementary file 1 — Supporting File: bimj70134‐sup‐0001‐DataCode.zip. [file BIMJ-68-e70134-s001.zip › MissingDataTmle/Simulation/design-based/Results/reference/quick/figures/Figure_6.png]

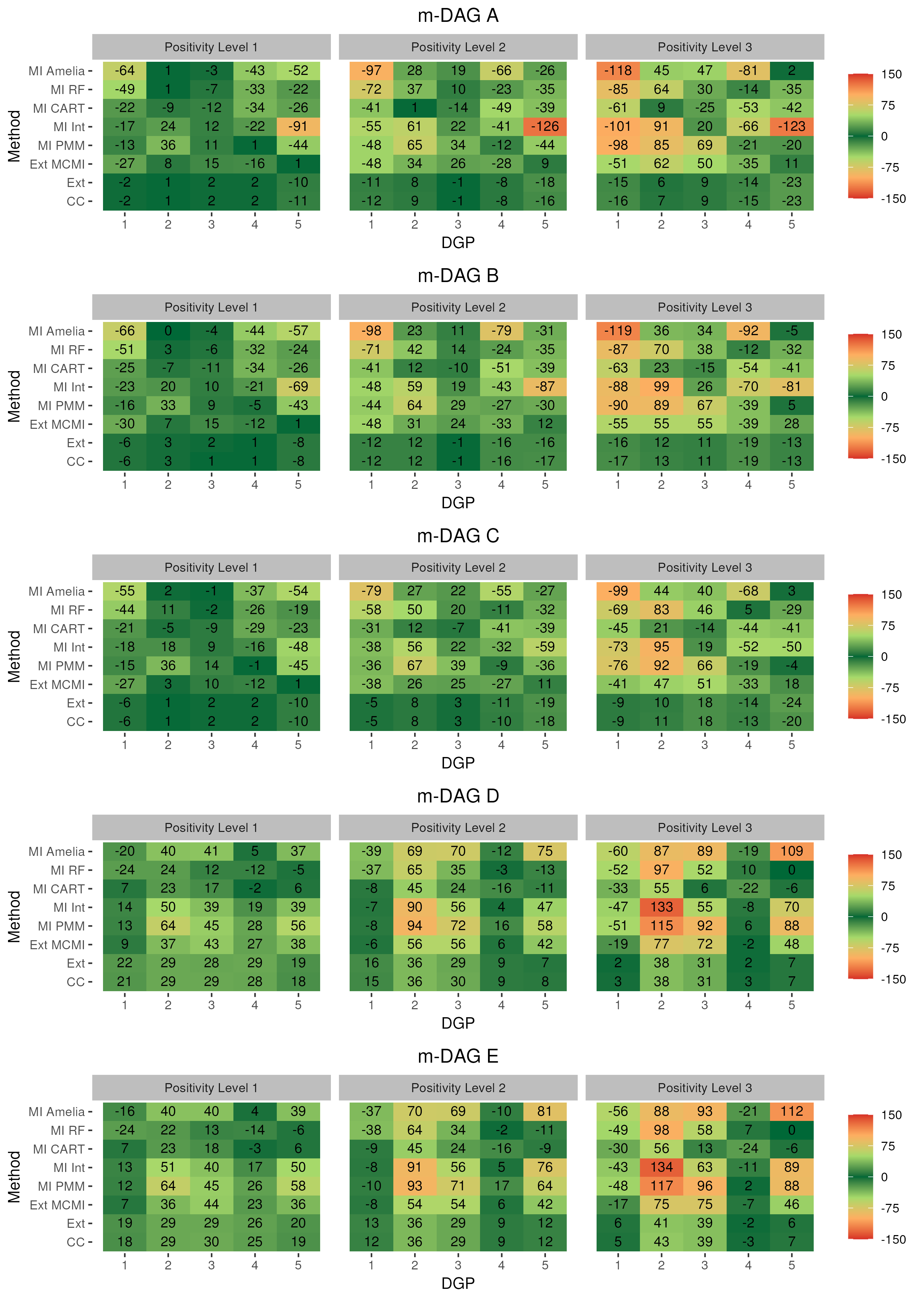

Supplement: Supplementary file 1 — Supporting File: bimj70134‐sup‐0001‐DataCode.zip. [file BIMJ-68-e70134-s001.zip › MissingDataTmle/Simulation/model-based/Results/reference/full/figures/Figure_3.png]

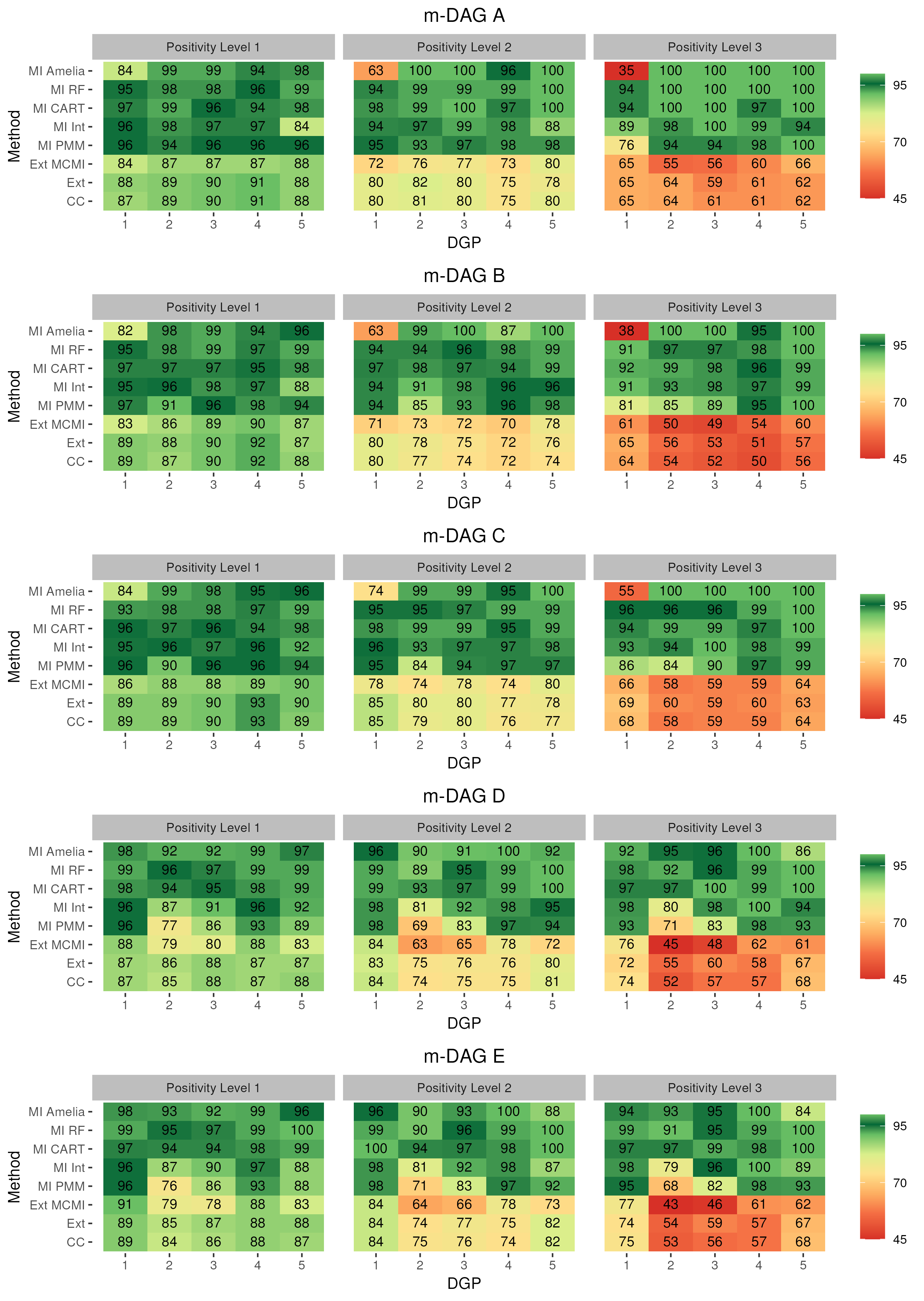

Supplement: Supplementary file 1 — Supporting File: bimj70134‐sup‐0001‐DataCode.zip. [file BIMJ-68-e70134-s001.zip › MissingDataTmle/Simulation/model-based/Results/reference/full/figures/Figure_4.png]

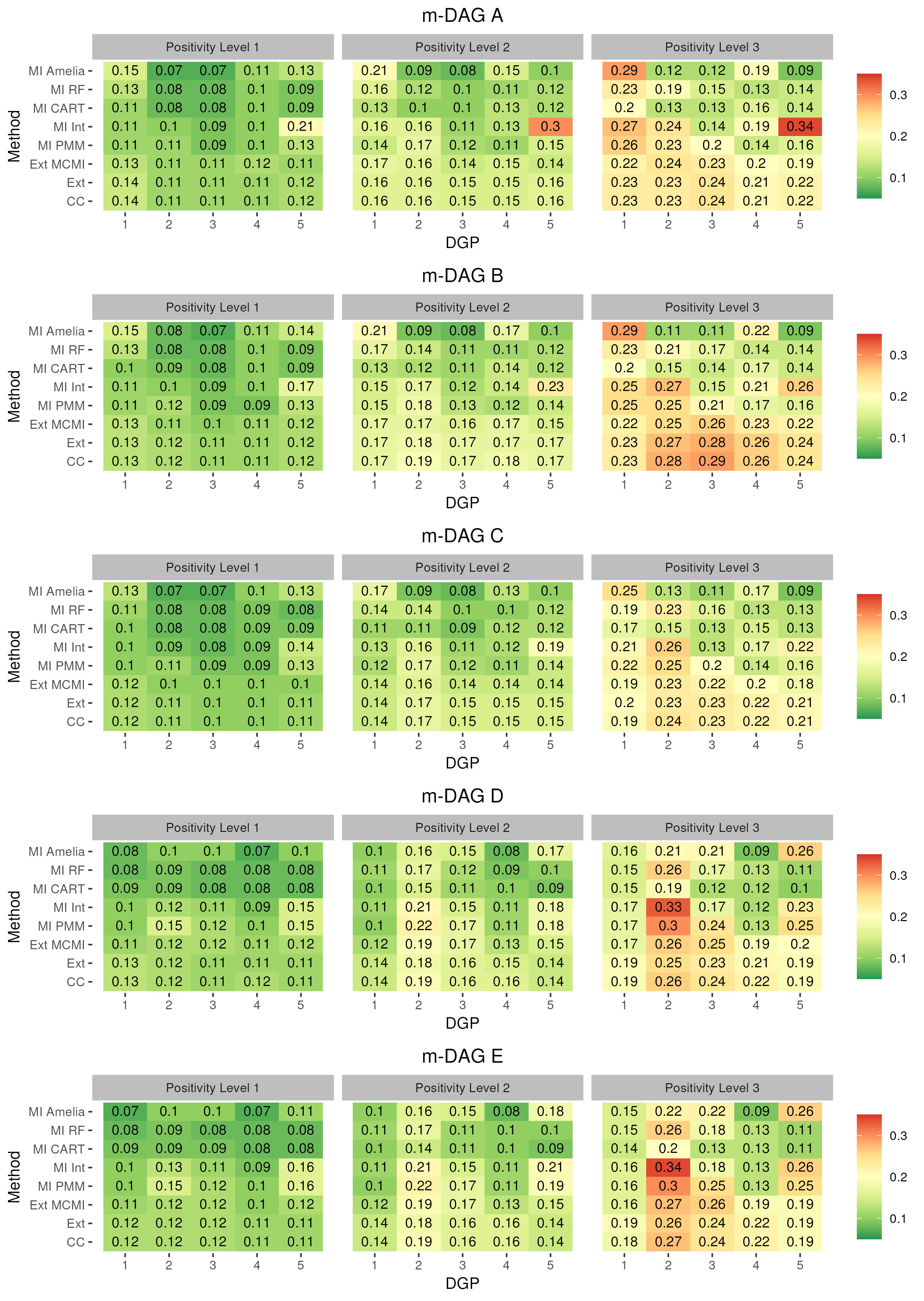

Supplement: Supplementary file 1 — Supporting File: bimj70134‐sup‐0001‐DataCode.zip. [file BIMJ-68-e70134-s001.zip › MissingDataTmle/Simulation/model-based/Results/reference/full/figures/Figure_5.png]

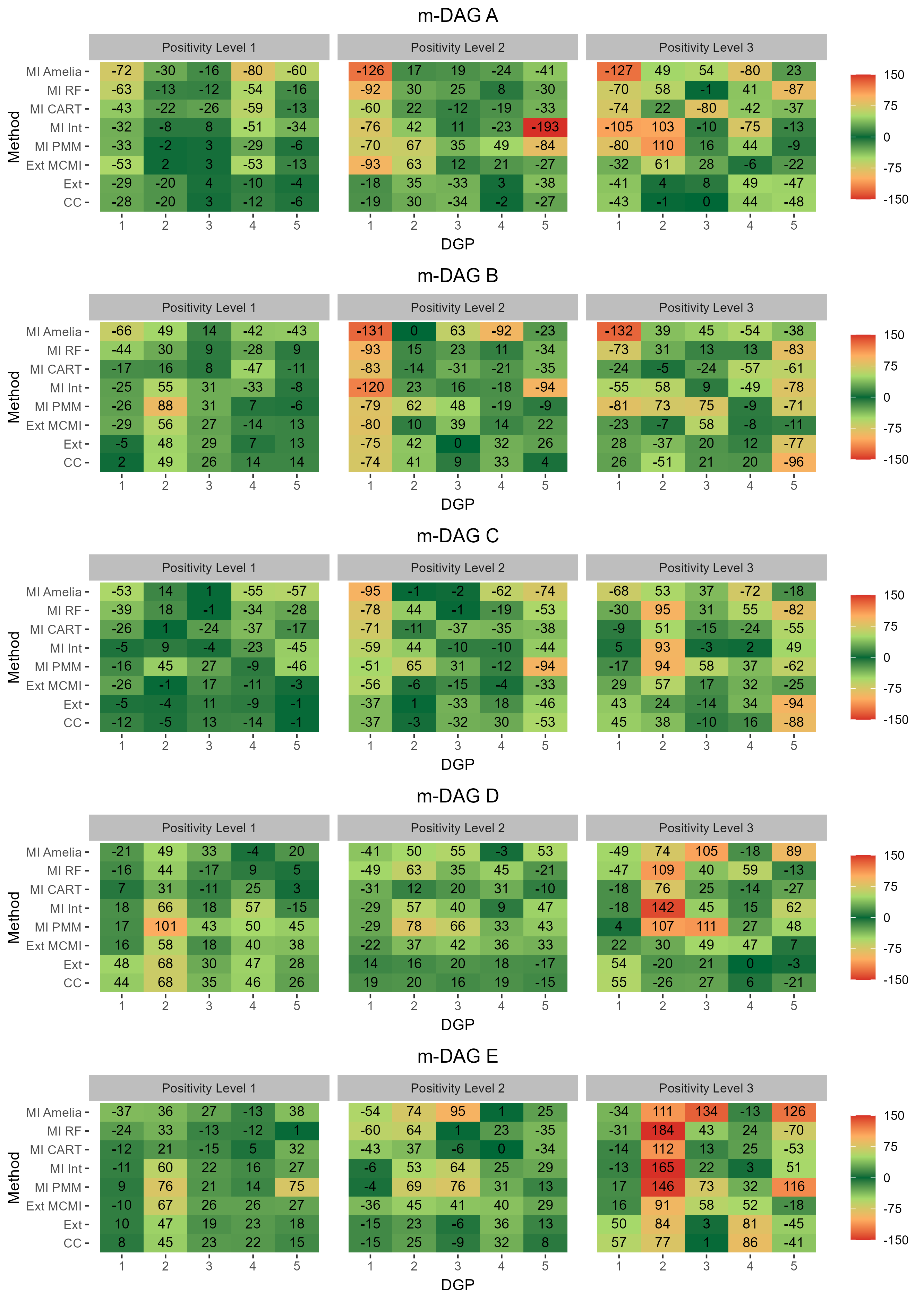

Supplement: Supplementary file 1 — Supporting File: bimj70134‐sup‐0001‐DataCode.zip. [file BIMJ-68-e70134-s001.zip › MissingDataTmle/Simulation/model-based/Results/reference/quick/figures/Figure_3.png]

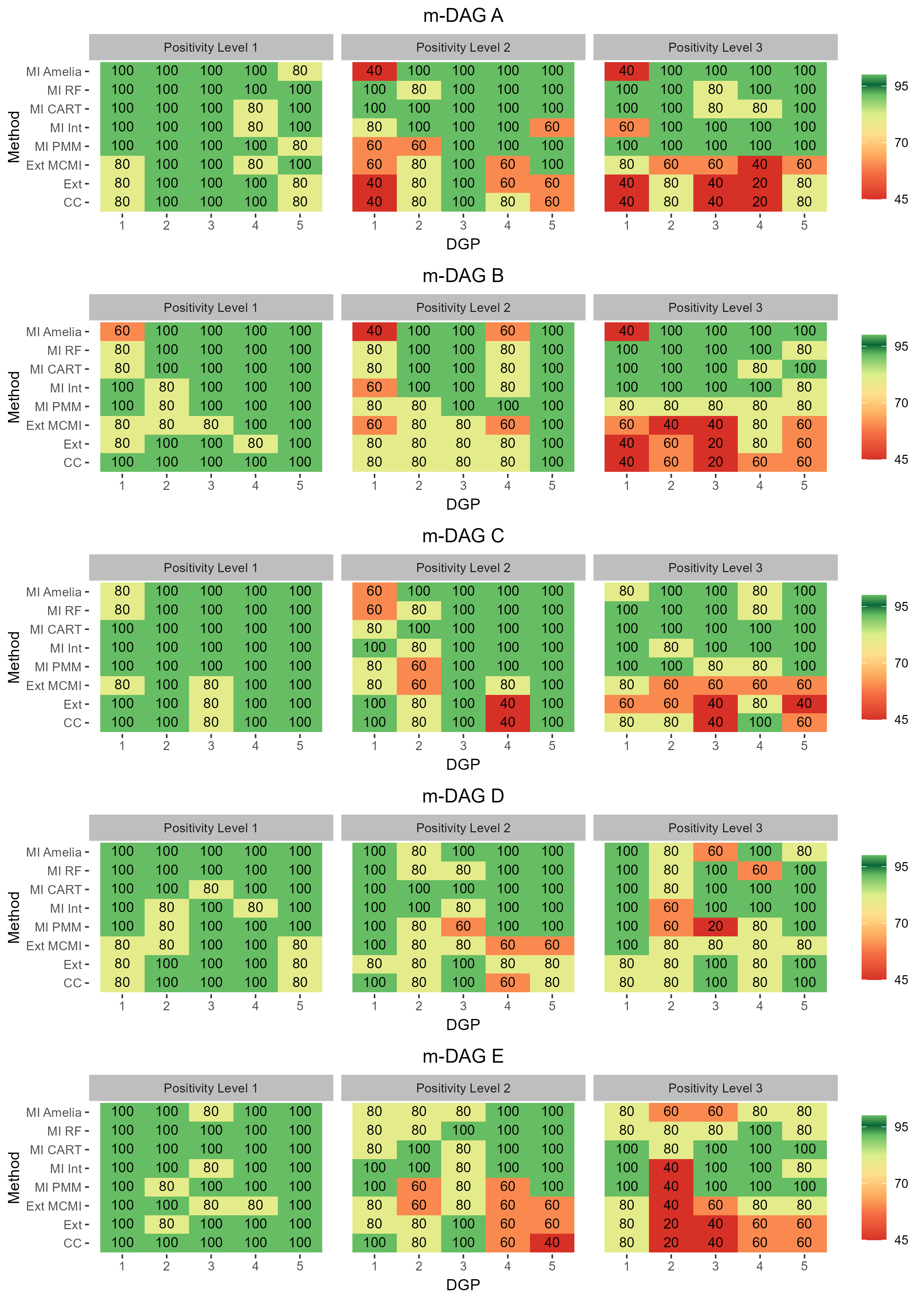

Supplement: Supplementary file 1 — Supporting File: bimj70134‐sup‐0001‐DataCode.zip. [file BIMJ-68-e70134-s001.zip › MissingDataTmle/Simulation/model-based/Results/reference/quick/figures/Figure_4.png]

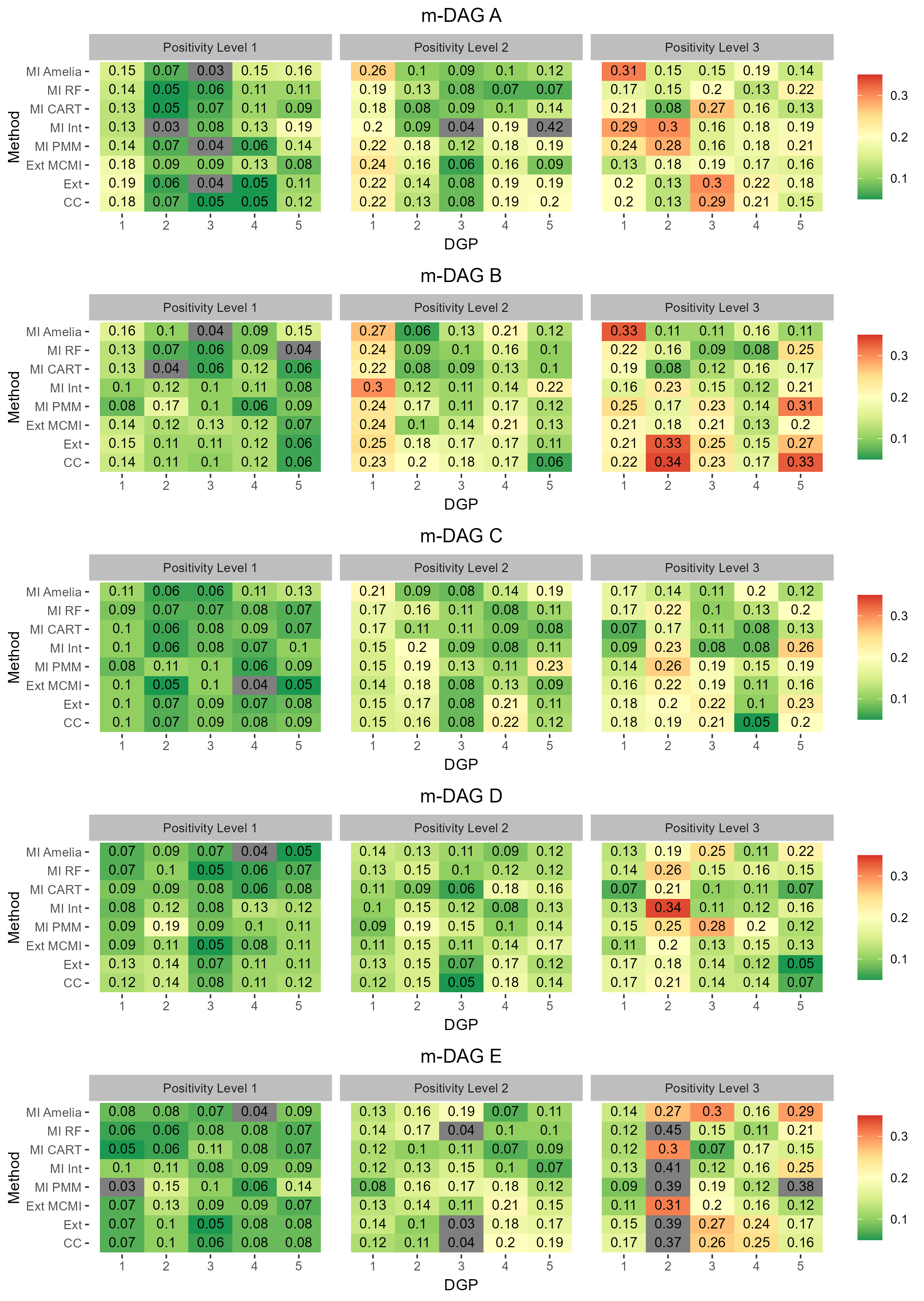

Supplement: Supplementary file 1 — Supporting File: bimj70134‐sup‐0001‐DataCode.zip. [file BIMJ-68-e70134-s001.zip › MissingDataTmle/Simulation/model-based/Results/reference/quick/figures/Figure_5.png]
